# C-Reactive Protein Levels in Pregnancy

**DOI:** 10.1289/ehp.1205429

**Published:** 2012-08-31

**Authors:** Luis Miguel Blasco

**Affiliations:** Unidad de Alta Resolución Hospitalaría, Hospital Marqués de Valdecilla, Santander, Spain, E-mail: grullus99@yahoo.es

van den Hooven et al. (2012) found a nonsignificant association between high levels of maternal and fetal C-reactive protein (CRP) and exposure to air pollution when they examined the correlation of CRP levels with inflammation and obstetric morbidity. The authors reported that elevated fetal CRP levels at delivery were associated with higher long-term average maternal exposure to PM_10_ (particulate matter ≤ 10 µm in aerodynamic diameter) and NO_2_ (nitrogen dioxide). Other studies have reported that neither preeclampsia (Kristensen et al. 2009) nor pregnancy loss (Boggess et al. 2005) is associated with a systemic inflammation as reflected by CRP levels. However, van den Hooven et al. (2012) insisted that exposure to air pollution may lead to systemic inflammation in pregnancy. Although this statement is defensible, the confounding results regarding CRP levels should be clarified.

CRP is accepted as a good marker of acute inflammation, particularly within infection, but its value in chronic inflammation depends on the inflammation pathway involved and the underlying process. In an examination of autoimmune inflammatory responses triggered by the indoor environment in sick buildings, CRP was < 0.1 mg/dL (normal range, 0.1–0.5 mg/dL) in 27% of patients (Blasco 2011). Interestingly, 13% of patients had suffered miscarriages. CRP may be low or typically very low during a flare-up of some connective tissue disorders, such as systemic lupus erythematosus (SLE) or undifferentiated connective tissue disease. The erythrocyte sedimentation rate more accurately reflects SLE disease activity in patients without associated infection. Therefore, the presence of normal or low CRP levels does not guarantee the absence of inflammation or a positive pregnancy outcome. It would be interesting to assess possible individual immune susceptibility markers and other markers, such as autoantibodies or tumor necrosis factor α, in future studies of systemic inflammation induced by air pollutants during pregnancy.
